# An Exploratory Study on the Development of a Pure Dairy Product Emotion Scale (PDPES): A Study of Milk Consumers in China

**DOI:** 10.3390/foods14050827

**Published:** 2025-02-27

**Authors:** Kui Zhong, Ting Wen, Bolin Shi, Houyin Wang, Zhicong An, Lei Zhao, Hongliang Li

**Affiliations:** 1 Agriculture and Food Standardization Institute, China National Institute of Standardization, Beijing 102200, China; zhongkui@cnis.ac.cn (K.Z.); shibl@cnis.ac.cn (B.S.); wanghy@cnis.ac.cn (H.W.); 2 Key Laboratory of Food Sensory Analysis, State Administration for Market Regulation, Beijing 102200, China; 3 National Enterprise Technology Center, Inner Mongolia Mengniu Dairy (Group) Co., Ltd., Huhhot 011500, China; wenting1@mengniu.cn (T.W.); anzhicong@mengniu.cn (Z.A.); 4 Mengniu Hi-Tech Dairy Product Beijing Co., Ltd., Beijing 101100, China

**Keywords:** pure dairy product, consumer test, emotion scale, reliability and validity

## Abstract

Emotions evoked by food are an important factor that strongly influences the predictive power of consumer acceptance and consumption behavior when choosing food. The emotion questionnaire is an efficient measurement tool that describes the emotions evoked by different foods on the market. However, there is rarely a list of emotion terms for specific foods, such as the pure dairy products on the market. The aim of this study was to develop an emotion scale to measure emotion responses for pure dairy products and to investigate its application in consumer testing. A total of 1340 Chinese subjects (67% female, 18~45 years old, consumers of pure milk) participated in the study. The study began with the screening and identifying of emotion terms for pure dairy products and developed a proposed pure dairy product Emotion Scale (P-PDPES) with 50 emotion terms. Subsequently, the structure and emotion terms of the P-PDPES were determined using EFA and a final pure dairy product Emotion Scale (PDPES) with 33 emotion terms was obtained. The results showed that the PDPES has good validity and reliability. Moreover, the PDPES was used in a consumer test to describe the emotions towards the commercial pure dairy products. This PDPES discriminated well the emotions evoked by different pure dairy products and also indicated a good relationship between the model factors and the liking scores of milk products. To summarize, PDPES is a suitable measurement tool for assessing the emotions to pure dairy products.

## 1. Introduction

Milk is a highly valuable and popular food, consumed by 80% of the world’s population [1]. It is known as nature’s most complete food and plays an important role in human nutrition. It provides high-quality protein, calcium, phosphorus, vitamin A, vitamin D and vitamin B2 [2]. As consumption increases and health awareness rises, the consumption of milk and dairy products has gradually increased worldwide in recent years and is also growing rapidly in China. Milk consumption per person was about 43 kg in 2022, an increase of 48.3% compared to 2010. In 2022, the production of milk and dairy products in China reached 40.27 million tons and 31.18 million tons, an increase of 6.6% and 2%, respectively, compared to 2021. As suggested by the Dietary Guidelines for the Chinese residents, an adult should consume no more than 300 g of milk or dairy products per day.

Ultra-high temperature (UHT) milk is currently representing the largest category in the Chinese dairy market. Due to increasing product innovation, changing dietary habits and the demand for healthy products, the categories of milk and dairy products on the market have become more numerous in recent years. These include pasteurized milk, whole milk with a higher protein content and calcium content, low-fat and skimmed milk and hydrolyzed milk that is lactose free [3]. These milk categories offer a variety of sensory flavors and more nutrients and vitamins to meet individual consumer needs (e.g., for people with weight-loss needs or lactose intolerance) and increase product acceptance.

The pair/rank preference method and the acceptability scale are the traditional and classical hedonic test methods in the current study [4]; however, they are still considered to be less efficient and accurate in predicting consumer behavior in food choices [5,6,7,8]. In recent decades, many novel consumer perception measurement methods have been developed that provide additional information to the product acceptance prediction, including information on sensory attributes and emotional responses to food [6,9]. Especially there has been particular interest in the emotions associated with the hedonic test and food consumption behavior in recent years, which are thought to greatly increased the impact of the predictive power of food choice [7,8,10]. Therefore, the question of how to accurately measure the emotions evoked by food in relation to food choice behavior has become a very important and interesting study. The evaluation of emotional measurements in food-related consumer research usually be divided into two types: the explicit method and implicit method [10].

The James–Lange theory of emotions suggests that emotional experience arises from the perception of an individual’s physiological reactions, which can manifest themselves in overt behaviors. Emotions can be measured along two dimensions: Valence (i.e., the positivity or negativity of the emotion) and Arousal (i.e., the intensity of the emotion). The explicit method is a self-reported questionnaire method in which respondents are asked to describe their emotional feelings related to food consumption [10]. General would provide the consumer with a list of emotion terms and ask them to select the applicable terms, of which EsSense 39 [11] and EmoSensory wheel [5] and PANAS [12] are most commonly used in the food and beverage. In addition, cartoon characters [13] or emojis [14] are visual self-reported methods that have also been used instead of verbal terms in emotional measurement, which are easily used in no language or other languages, so are more use for children or in cross-cultural consumer research [15]. Modern neuroscientific research shows that the implicit processing of emotions is closely linked to certain brain regions such as the amygdala and the prefrontal cortex. These regions play an important role in the automatic processing of emotional stimuli, even when the person is not aware of the presence of emotions. The implicit method is a measurement of the emotional response elicited by the food itself, in an automatic and unconscious process. Therefore, these measurements are not self-reported and are not be influenced by the consumer’s cognition, expression and experience [16]. The implicit reactions can be measured by eye-tracking measurements, facial expression analysis and physiological measurements (e.g., ECG/EDA/EEG) [17]. About 55.4% of studies only used cognitive explicit instruments, which were published between 2006 and 2021 (median = 2018), while 23.3% of studies used a combination of measures and were published between 2017 and 2019 (median = 2019) [8].

Both questionnaires and implicit methods offer the additional value in research of food choice; however, some reports pointed out that explicit methods are better suited to evaluate the emotion perception of food experiences [10]. The addition of implicit methods has only a minor influence on the evaluation of product differentiation [18,19]. Some implicit methods (e.g., facial expression analysis) usually focus on a small number of (mostly negative) emotions [20]. When it comes to evaluating food in a commercial context, most emotional responses in food experiences are positive [11]. Especially in the evaluation of commercial foods, the questionnaire method can provide more comprehensive emotion terms that could be better describe and identify the difference in positive emotional experiences [10]. Furthermore, questionnaires with emotions in the consumer test are user friendly, quick and easy to complete.

Emotion terms in the emotion questionnaire method are very important for the accuracy of the measurement. Currently, there are several questionnaire methods that use common emotion terms, such as EsSense Profile^®^, EmoSensory^®^ and others. These emotion terms have been applied in different food categories to describe the different emotions of different foods on the market. However, there is rarely a list of emotion terms for specific foods, such as the pure dairy products (PDPs) in the market. Overall, the objective of this study is to develop a scale method to quantitatively measure the emotion responses evoked by PDPs. This method is mainly used for the commercial food and the respondents are the consumers of PDPs. In particular, the current research aims at the following processes: (a) screening and identifying of emotion terms evoked by a pure dairy product in consumers; (b) development of an emotion scale for pure dairy products (PDPES) with good validity and reliability for evaluating emotions in PDPs; and (c) identification of the PDPES and application of the method in consumer test.

## 2. Materials and Methods

### 2.1. Participants

The data were collected by means of a questionnaire survey using Computer-Assisted Personal Interviewing (CAPI) and a consumer Central Location Test (CLT). A total of 1340 Chinese subjects (67% female, 18~45 years old; individuals with lactose intolerance were not included) participated in this study. These participants came from different regions of the country. The study was divided into four sections and the descriptive statistics of the participants in each study section are shown in Table 1. The surveys in Studies 1 to 3 were conducted by Credamo (https://www.credamo.com, accessed on March 2024) and respondents were recruited from their database, with the screening condition being consumption of pure milk and yoghurt at least twice a week. Respondents of Study 4 were recruited from the China National Institute of Standardization consumer database, and the screening condition is the same as in Studies 1 to 3. The target for questionnaire length was not to exceed 10–15 min for an internet survey and <60 min for a consumer CLT. The study was conducted in accordance with the Declaration of Helsinki. All subjects signed an informed consent form before the study and paid after the test. This survey was approved by the Ethics Committees of Tsinghua University.

### 2.2. Procedure and Methods

Table 2 provides an overview of the four study sections and their contributions to the assessment of the validity of the PDPES.

**Study 1.** The aim of Study 1 was to screen and identify the emotion terms that are highly correlated with the pure dairy product. The list of emotions to be included in this study came from three sources: existing emotion terms, an industry survey conducted by Essence Corporation and internet feedback from consumers. Existing emotion terms include the EsSense Profile (39 terms), MMR (30 terms), EmoSensory (19 terms), PrEmo (14 terms) and PANAS (20 terms). Consumer feedback was collected from thousands of consumers on the internet. A total of 88 terms from existing emotion questionnaires and 43 terms from the essence corporation survey and consumer feedback are listed in Supplementary Table S1.

In Study 1, an internet survey was conducted using a check-all-that-apply (CATA) method to identify the terms stimulated by PDPs. Respondents (n = 600, 67% female, 18–45 years old) were asked to describe their daily consumption of PDPs. They were then presented with an emotion questionnaire and asked to indicate all emotions in the options describing how they felt when consuming the PDPs (Supplementary Figure S1). In total, 131 terms were randomly divided into four questions and the order of the four questions and the emotion terms in each question was randomized. Only terms with a frequency of more than 20% were selected for the study. Subsequently, these terms were evaluated individually and/or grouped into 2–3 terms based on the similarity of their definition (Xinhua dictionary of the Chinese edition was used to identify the grouping). Finally, 50 emotions were identified and used to develop the proposed pure dairy product Emotion Scale (P-PDPES).

**Study 2.** The aim of Study 2 was to develop a definitive pure dairy product Emotion Scale (PDPES) with good validity. This study was divided into two sub-studies, Study 2A and Study 2B.

***Study 2A.*** Purpose of Study 2A was to assess the construct validity of the P-PDPES (50 emotion items). Respondents (N = 300, 67% female, 18–45 years old) completed the emotion questionnaire, the questions of which were identical to those in Supplementary Figure S1. They were also asked to rate each emotion according to the degree of agreement with the emotion on a five-point Likert scale from 1 = definitely disagree to 5 = definitely agree. The number of emotions in the list was 50 and the order of the 50 emotions was randomized for each respondent. The data were then assessed using an Exploratory Factor Analysis (EFA) and then Bartlett’s test for sphericity and Kaiser–Meyer–Olkin (KMO) were calculated [21,22]. The factor loading of each emotion term and minimize between-item variance on the factors were estimated. The determination criteria for the number of potential underlying factors were applied as follows: Eigenvalues > 1, common factors > 0.3, factor loadings > 0.4 and items loading on two or more factors > 0.3 (and the difference between two factor loadings is no more than 0.3) and no fewer than three variables in each factor [23]. According to the above criteria, the variables that do not meet the criteria are reviewed and deleted one by one. Finally, the final emotion scale was created with 33 emotions.

***Study 2B.*** Purpose of Study 2B was to further assess the construct validity of the identified emotion questionnaires for PDPs (33 emotions) using Confirmatory Factor Analysis (CFA). A total of 500 respondents (67% female, 18–45 years old) completed the questionnaire, with the order of emotions randomized for each respondent. They were asked to rate each emotion term according to the degree of agreement with the term on a five-point Likert scale from 1 (definitely disagree) to 5 (definitely agree). The model was estimated using maximum likelihood with robust standard errors. The model parameters including Chi-square of the freedom ratio, comparative fit index (CFI), Tucker–Lewis index (TLI), root mean square error of approximation (RMSEA) and standardized root mean square residual (SRMR) are used to assess the model fit [24]. The average Variance Extracted (AVE) was estimated to assess the convergent validity and discriminant validity between the dimensions of the PDPES. In addition, the composite reliability was calculated to assess the convergent validity. Cronbach’s alpha coefficient, average inter-item correlation (AIC), composite reliability and maximum reliability were calculated to assess the internal consistency reliability of the scale [25].

**Study 3.** The aim of Study 3 was to assess test-retest reliability. In total, 50 respondents, randomly selected from the participants of Study 2B, were asked to complete the same scale again. The interval between the two tests was approximately 4 weeks, which is consistent with previous reports on the development of scale. Pearson’s correlation coefficient between the two tests was used for the test–retest reliability of the scales.

**Study 4.** The aim of Study 4 was to evaluate the application effect of the validated PDPES in real pure dairy products using a CLT test. Five commercially available pure dairy products were used for this study, including four whole milk products (two UHT milk products, one pasteurized milk product and one lactose-free milk product) and one low-fat milk product. These products were purchased from local Chinese supermarkets and the main ingredient information (fat, protein, carbohydrate and sodium content) was obtained from the nutritional composition labels on the product packaging (Supplementary Table S2). These milk products were produced domestically and the production date was May 2023. Study 4 was conducted in July 2023 and lasted 1 month.

The preparation of the milk sample was completed within 24 h prior to the experiment. The 30 mL milk sample was placed in a separate 50 mL PET tasting cup with a lid and a randomized three-digit label. Samples were presented to each respondent in a randomized order. In total, 70 respondents (67% female, 18~45 years old; individuals with lactose intolerance were not included) were recruited to participate in this study. First, they had to complete an acceptability test of test milk sample using a nine-point hedonic scale (1 = dislike extremely, 2 = dislike very much, 3 = dislike, 4 = dislike slightly, 5 = neither like nor dislike, 6 = like slightly, 7 = like, 8 = like very much, 9 = like extremely). These respondents then completed an emotion perception test of this milk sample using the PDPES questionnaire, which was identical to that used in Study 2B. The order of the emotion terms was randomized for each respondent. AppSense V9.0 software was used to conduct the test and record consumer responses. Five milk samples were presented simultaneously in random order, while the temperature in the test room was kept at around 25 °C.

### 2.3. Statistical Analysis

Descriptive statistics, EFA, CFA and test-retest reliability were all performed with SPSS 25.0 (SPSS Inc., Chicago, IL, USA). For EFA, the criterion that the data are suitable for EFA is the Bartlett’s test for sphericity (*p* < 0.05) and higher KMO value (>0.6). For CFA, the requirements for the indices for a good model fit are as follows: *X*^2^/*df* ≤ 5), CFI ≥ 0.90, TLI ≥ 0.90), SRMR < 0.08 and RMSEA < 0.08. An average inter-item correlation of at least 0.50 was regarded as good; a Cronbach’s alpha > 0.70 and reliability coefficients of 0.70 or higher were considered to indicate good reliability [26,27]. To establish convergent validity, it was assumed that the average Variance Extracted should be greater than 0.5 and the composite reliability greater than the respective average Variance Extracted (AVE) [28].

## 3. Results

### 3.1. Emotion Term Screening and Identification

**Figure 1:**
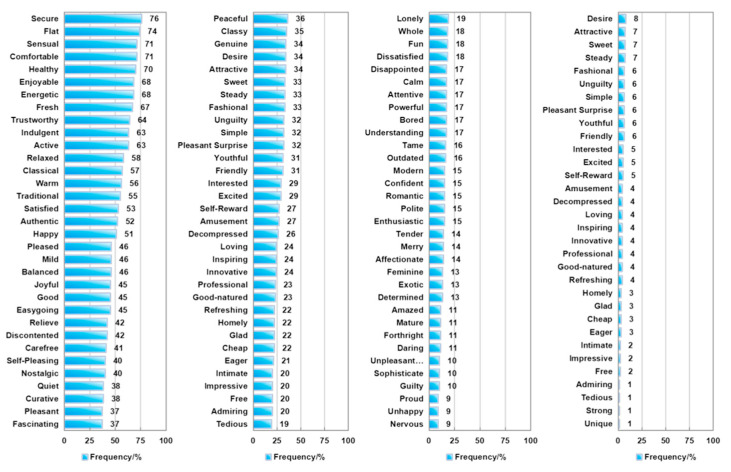
Selected frequency of emotion term for fluid milk products.

The emotion terms were determined by describing the respondent feelings when consuming the pure dairy products on a daily basis using an internet survey. Figure 1 shows the selected results of the emotion terms in Study 1. Of the 132 emotion terms evaluated, 18 terms were selected for more than 50% and 47 terms for 20% to 50%. The five terms with highest selected frequency were secure, flat, sensual, comfort and health, which were selected by more than 70%. In addition, terms such as enjoyable, energetic, fresh and trustworthy were selected with a frequency of more than 60%. Almost all terms with a frequency above 50% are positive emotion terms or more positive emotion terms, such as comfort, good, secure, energetic and so on. Parts of the terms selected between 20% and 50% are no clear classification, such as eager, mild, polite, quiet, surprised, wild, young and so on. Most of the negative emotions were selected at less than 20%, and mainly at less than 10%, except discontented (42%) and cheap (22%).

**Figure 2:**
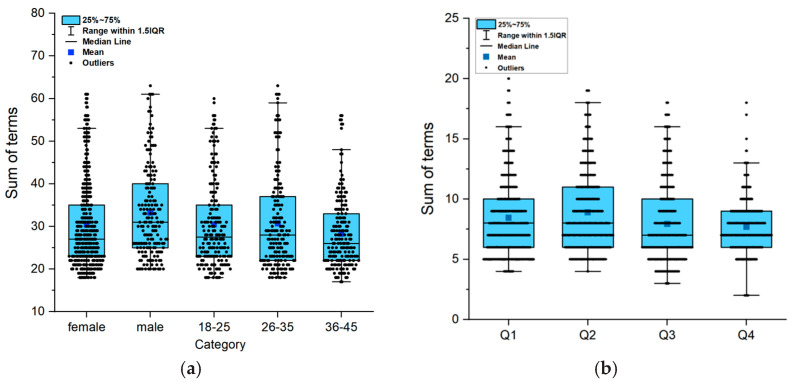
Statistics of the number of selection terms (n = 600). (**a**) Total number of selection terms, (**b**) Number of selection terms in each question.

Gender and age had no significant influence on the total number of terms selected and the mean value was between 30.6 and 35.8 (*p* > 0.05). As shown in Figure 2, the order of the four questions also had no significant influence on the number of selected terms (*p* > 0.05). There was also no significant difference in the frequency of selected terms (>20%) by gender, while group C (36~45 years) showed a significant difference to group A (18~25 years) and group B (26~35 years) (*p* < 0.05).

A total of 65 terms with a frequency of more than 20% were selected. These terms were then processed on the basis of a semantic analysis, including the merging of synonyms and the elimination of words that do not contain emotions. Only one term remained, e.g., sensual (71%) and sensual (63%), relaxed (58%), relieved (42%) and decompressed (26%), self-pleasing (40%) and self-reward (27%). In addition, the terms healthy, fresh, balanced, innovative and professional were deleted. Finally, 50 emotion terms were proposed for pure dairy products and listed in Table 3.

### 3.2. Development of Emotion Terms

**Construct validity.** The data collected from respondents in Study 2 (N = 300) were analyzed using CFA. The results showed that this questionnaire with the 50 proposed emotion terms did not have good construct validity. Therefore, an EFA was conducted to determine the factor structure. Fifty emotion terms were factor analyzed using PCA with varimax rotation. Six factors with an eigen-root > 1 were identified and the explanatory rate of the cumulative variance was 53.45%. According to the KMO value (KMO = 0.849) and the Bartlett’s test for sphericity (*X*^2^ = 4798.46, *p* < 0.001), the data set was suitable for EFA. However, the terms, including nostalgic, youthful, fashionable, toneless, lonely, discontented, cheap, bored, whole and carefree with common factor < 0.3 or with factor loading < 0.4, appeared to be poor and therefore these 10 terms were eliminated.

Factor analysis was then performed again with promax rotation in order to analyze the factor structure. Five factors with an eigen-root > 1 were obtained and explanatory rate of the cumulative variance was 51.70%. The KMO value was 0.837 and Bartlett’s test for sphericity (*X*^2^ = 4528.67, *p* < 0.001) was carried out. The terms, including attentive, free, traditional, fascinating, intimate, strong and excited, were found to be poor, with a factor loading < 0.4 or with two similar factor loadings on two factors where the difference between the two factor loadings was no more than 0.05 and therefore these seven terms were then eliminated.

Finally, the third FA was performed with promax rotation, the KMO value was 0.815 and Bartlett’s test of sphericity was statistically significant (*p* < 0.001), indicating that the four-factor structure was appropriate. The eigenvalues of the four factors were 8.621, 3.348, 1.994 and 1.494. The explanatory rate of cumulative variance reached 51.32%. Table 4 presents strong factor loading of 33 terms ranging from 0.409 to 0.886 (factor loading > 0.4 can be considered good) [29]. The four factors have a positive significant relationship (Table 5). *Factor 1* contains 13 terms (α = 0.843); this factor examined the explicit cognition in response to what the pure dairy product elicits. *Factor 2* contains six terms (α = 0.766); this factor examined the cognitive emotions that the pure dairy products aim to convey to the consumer. *Factor 3* contains nine terms (α = 0.803); this factor examined the level of acceptance that the consumer perceives from the pure dairy product. *Factor 4* contains five terms (α = 0.715); this factor analyzes the emotion response, which cannot be clearly divided into positive or negative emotions.

CFA with the MLM method was used in the Study 2A and 2B and the results are shown in Table 6. A poor fit was observed for the proposed emotion scale: *χ*^2^/*df* = 2.63; CFI = 0.72, TLI = 0.66, RMSEA = 0.08, SRMR = 0.08. However, a good fit was observed for the final emotion scale: *χ*^2^/*df* = 2.16; CFI = 0.95, TLI = 0.93, RMSEA = 0.07, SRMR = 0.04. Therefore, the final PDPES with 33 terms has good construct validity.

**Convergent and Discriminant validity.** Convergent and discriminant validity of the final PDP Emotion Scale was assessed. The composite reliabilities of the four factors were between 0.689 and 0.859, which were higher than 0.7 and indicating good convergent validity. The results of the correlations and the square root of AVE are shown in Table 7. The values of square root of AVE were between 0.566 and 0.699 and the correlations of the factors were between 0.111 and 0.664. A latent dimension should better explain the variance of its own indicator than the variance of other latent dimensions. Therefore, the square root of the AVE of each dimension should have a greater value than the correlations with other latent dimensions [28]. In this study, the square root of AVE of each factor was higher than the correlation between that factor and the other factors, indicating good discriminant validity.

**Reliability Analysis.** As shown in Table 8, the final PDP Emotion Scale had a good internal consistency of the total score (0.875) and the four factors (0.715~0.843). The reset reliability had a similar result, namely the total score (0.663) and four factors (0.602~0.703).

### 3.3. Consumer Test

**Emotion Test.** The emotional response to five milk products was measured using the PDP Emotion Scale and the results are shown in Table 9.

**Figure 3:**
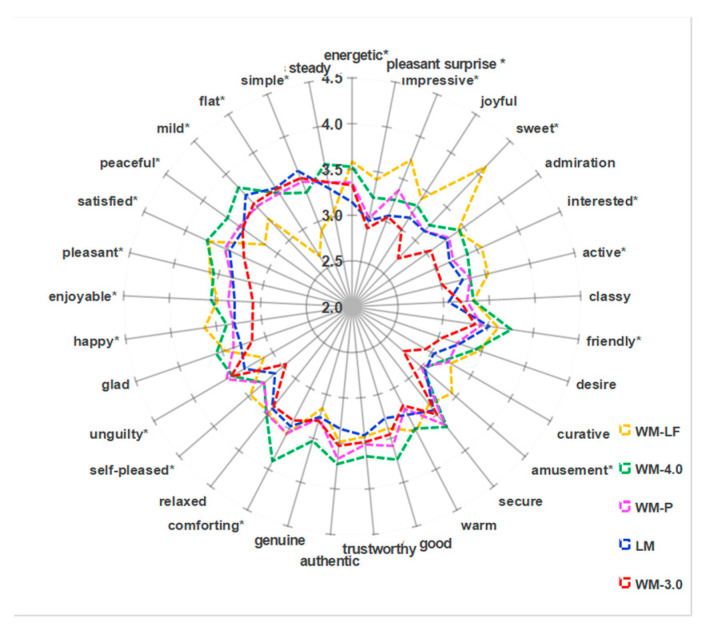
Emotion profile of milk products (n = 70). (Note: * Indicates a significant difference at *p* < 0.05.)

The mean values of four emotion factors were between 3.26 and 3.45, indicating that these products do indeed evoke emotions in the PDPES. Kruskal–Wallis test was performed for the emotion intensity of the individual factors and significant differences were found between the milk samples for *factor 1* (Property Cognition), *factor 3* (Acceptance Appraisal) and *factor 4* (Neutral Property Cognition) (*p* < 0.05). For *factor 1* and *factor 3*, WM-4.0 and WM-LF had a significant higher emotion intensity than WM-3.0. In addition, WM-LF had significantly stronger emotion intensity on *factor 1* than LM and WM-LF had a weaker emotion intensity on *factor 4* than the other samples. Figure 3 presented the emotion profile (33 emotions) of five milk products. Of the 33 emotions, 22 terms showed significant intensity differences between five milk products (*p* < 0.05). The *t*-test results showed that WM-3.0 and LM had the lower intensity of most emotions (8 out of 13 emotions, 61.5%) of *factor 1*, WM-LF had the lowest intensity of most emotions (6 out of 9 emotions, 66.7%) of *factor 3* (except relaxed, unguilty and glad) and WM-LF had the lowest intensity of the all emotions (100%) of *factor 4*.

**Figure 4:**
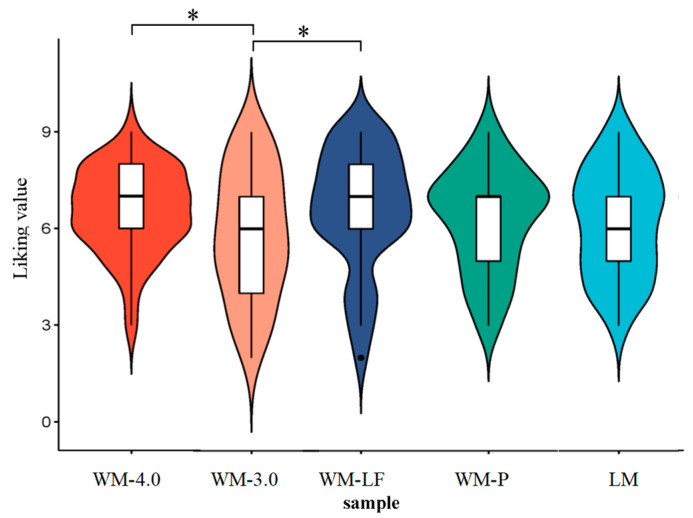
Hedonic test of milk products (n = 70) (note: * indicates a significant difference at *p* < 0.05).

**Hedonic Test.** As shown in Figure 4, the overall liking scores for the five milk samples were higher than 5 and ranged from 5.73 (like slightly) to 6.61 (like), indicating that these five milk products were preferred by the most consumers. The result of the *t*-test showed that WM-4.0 and WM-LF had significantly higher liking scores than WM-3.0 (*p* < 0.05). In addition, LM and WM-P showed no significant differences in liking scores compared to other WM products (*p* > 0.05).

Factor scores were correlated with the liking scores of five milk products with Spearman correlation analysis (Table 10). Significant correlations were observed between the liking scores and the factor scores. The liking scores of five milk products showed a higher correlation coefficient with *factor 3 *(Acceptance Appraisal) and a lower correlation coefficient with *factor 4* (Neutral Property Cognition).

## 4. Discussion

The aim of this study was to develop an emotion scale to measure emotion responses for commercial pure dairy products. The study began with the screening of emotion terms for pure dairy products and then developed a proposed pure dairy product Emotion Scale (P-PDPES) with 50 emotion terms. Subsequently, the structure and emotion terms of the P-PDPES were determined using EFA to obtain a final PDPES with 33 emotion terms. The PDPES were further assessed for construct validity and reliability and finally used in the consumer test to describe and discriminate the emotions of the pure dairy products.

In the final PDPES of 33 terms, there were no negative terms. In the first screening of emotions, the selected frequency of most negative terms was very low, focusing on less than 10%, with the exception of two terms such as discontented (42%) and cheap (22%). In addition, the EFA results of the P-PDPES also showed the low common factor (<0.3) or low factor loading (<0.4) of the terms, such as discontented, cheap and bored. This result was consistent with the previous report [10,30,31]. For most commercial foods, food consumers tend to have positive experiences with food and have stronger positive emotions than non-food consumers and the intensity of emotions increases with increasing frequency of food consumption [11,16]. In this study, the subjects were actual consumers of pure dairy products who consumed milk more than twice a week. The questionnaire asked them what emotions they felt about their daily milk consumption. Therefore, they rarely experienced negative emotions when drinking milk and rarely selected the negative emotions in the test.

Both explicit and implicit methods are effective tools for measuring emotions evoked by food and there have been many reports on this in recent years [8]. Compared to the implicit method, the questionnaire test contains a longer list of emotions and only a few negative emotions, which makes it possible to better represent the emotional response and avoid the loss of low-intensity emotions associated with eating behavior [19,32]. In addition, some reports have indicated that that the questionnaire method can provide more comprehensive emotion terms that can better describe and identify differences in positive emotional experiences [10]. For example, the scales of EsSense 39 (39 terms) and MMR (30 terms) have few negative terms that are commonly used to measure the emotional response to food [11].

Four factors were determined through EFA that correspond to the four different emotion dimensions of PDPES and the result of the CFA shows a good model fit. The four emotion dimensions are: Property Cognition (*factor 1*), which examines the emotions that a food’s property evokes in consumers; Quality Perception (*factor 2*), which assesses consumers overall perception of food quality; Acceptance Appraisal (*factor 3*), which measures the degree to which consumers accept the food; and Neutral Property Cognition (*factor 4*), which examines the quiet (calm) emotions that a food’s property evokes in consumers. The results of construct validity and reliability indicate a good convergent and discriminant validity and internal consistency, which further demonstrate the good stability of the four factors.

The terms in *factor 1* (Property Cognition) and *factor 4* (Neutral Property Cognition) are the emotions evoked by the natural properties of milk products, with the emotions in *factor 1* being positive emotions and *factor 4* being the neutral or not quite as positive emotions. Milk product contains nutrients that are suitable for humans in a natural form, such as proteins, polyunsaturated fatty acids, vitamins and minerals, which is an important part of a healthy and balanced diet [33]. The emotions of energetic, active and sweet are associated with these native, physicochemical and nutritional properties of milk products. The emotions of amusement, classy, admiration and impressive, which are associated with the socially relevant properties of dairy products [34,35]. Food choice behavior has multifactorial psychological reasons, including social, cognitive and affective [36,37]. The emotions in *factor 4* (i.e., peaceful, mild) are also considered as emotions that cannot be clearly categorized as positive or negative emotions [11].

A very interesting phenomenon is the emotion term “self-pleasing”, which played a role in the acceptance appraisal factor. This term was collected in the internet survey and showed a high selected frequency and factor loading. Self-pleasing is not a basic and traditional emotion term that has only emerged in recent years and describes an attitude of doing things to please oneself. It is also an important factor and a label that influences the food choices and consumption of Generation Z (people born between 1995 and 2009). This emotion terms means that every young person in Generation Z hopes to achieve a genuine sense of happiness by pleasing their true selves [38]. This emotional denial is also an important factor in product formulation and marketing strategy.

The final PDPESs were used to measure the consumer’s emotion responses to five different commercial milk products in a consumer test (N = 70). The consumer screening conditions were the same as for the internet survey. The natural property of milk products had a significant influence on the emotion response to factor of property cognition and acceptance of milk consumers (*p* < 0.05). Milk with high-protein content (WH-4.0) and lactose-free milk (WH-LR) had higher emotion scores on the factors of property cognition and acceptance appraisal than normal whole milk (WH-3.0) and low-fat milk (LM), while WH-LR had the lowest emotion score on the factor of quiet property cognition (*p* < 0.05). Specifically, for *factor 1*, both WH-LR and WH-4.0 had higher scores on the response intensities of terms energetic, pleasant surprise and friendly than LM, specifically WH-LR had the highest scores on the sweet, impressive, interested, active and amusement, and WH-4.0 had highest scores on comfort. Lactose intolerance is widespread in Asia and Africa. Lactozyme hydrolyzation technology is a modern processing technology that uses lactase to break down 90% of the lactose in milk into galactose and glucose without destroying other nutrients in the milk. This technology solves the problem of lactose intolerance in certain populations and ensures that the milk can be more easily absorbed by the human body [3]. Compared with the common whole milk, WM-LF evoked stronger emotion responses associated with positive and acceptance and decreased neutral emotion intensity. This was considered with the higher sweet sensory intensity of WM-LF. The component concentration of milk products can change the intensity of emotion associated with positive and acceptance; however, it cannot affect that of neutral emotion and quality-related perception.

One application of the emotion scale is that it provides more useful information about the acceptance of food. For example, it can show the difference in emotion response between two products with similar acceptance score. In this study, WM-LF and WM-4.0 had similar liking scores but a different emotion profiles. WM-P and LM had similar liking scores and similar intensity on most emotion attributes. In addition, acceptance was significantly related to four factors of PDPES, indicating a good application of PDPES in the acceptance study of commercial milk products.

## 5. Conclusions

The emotions evoked by food are an important factor influencing the acceptance and choice behavior of food. The emotion scale method has proven to be an effective tool for describing and identifying the emotions of different commercial foods. In this study, an emotion scale for commercial pure dairy products (PDPES) was developed, which shows good validity and reliability. The PDPES include 33 emotion terms and measure the milk-evoked emotions from four factors: it measures the emotions that consumers have in milk products by Property Cognition (*factor 1*), Quality Perception (*factor 2*), Acceptance Appraisal (*factor 3*) and Neutral Property Cognition (*factor 4*). This emotion scale demonstrated good emotion attributes and is able to explain and discriminate between different commercial milk products. In addition, the four factors all have significant correlations with liking scores of five commercial milk products. Therefore, the PDPES is a useful tool to evaluate the emotions of commercial milk products. In addition, the PDPES is not applicable to consumers who do not drink the milk product, as the scale does not include negative emotions. Future research will focus on analyzing the differences in emotional responses to dairy products among individuals with different characteristics (such as region, occupation and income) and on correlation analysis of self-reported PDPES data measured by a self-reported method with emotional data measured by implicit methods.

## Figures and Tables

**Table 1:** Descriptive statistics of participants in this study.

Study	Number of Participants (Male/Female)	Age			Mean Age	Drinking Frequency	
		18–25	26–35	36–45		Pure Milk	Yogurt
Study 1	600 (200/400)	200	200	200	35.4 ± 5.9	>2/week	>2/week
Study 2A	300 (100/200)	100	100	100	37.4 ± 7.8		
Study 2B	320 (107/213)	104	108	108	37.9 ± 5.5		
Study 3	50 (17/33)	16	16	18	32.9 ± 3.4		
Study 4	70 (23/47)	22	24	24	31.8 ± 6.8		

**Table 2:** Overview of the studies included in the development of PDPES.

Study	Survey Mode	Study Method	Purpose
Study 1	Internet survey	CATA/Written stimuli	Preliminary screening of terms
Study 2A	Internet survey	P-PDPES/Written stimuli	Content validity (EFA)
Study 2B	Internet survey	PDPES/Written stimuli	Construct validity (CFA)Convergent/Discriminant validity
Study 3	Internet survey	PDPES/Written stimuli	Reliability
Study 4	CLT	Hedonic test/PDPES/pure dairy product stimuli	Scale application/validation

**Table 3:** Emotion terms of the proposed pure dairy product Emotion Scale (P-PDPES).

No.	Terms		No.	Terms		No.	Terms	
	English Words	Chinese Words		English Words	Chinese Words		English Words	Chinese Words
1	Active	有活力的	18	Fashionable	时尚的	35	Pleasant surprise	惊喜的
2	Admiration	欣赏赞美的	19	Flat	平淡的	36	Relaxed	放松轻松的
3	Amusement	娱乐消遣的	20	Free	自由的	37	Satisfied	令人满意的
4	Attentive	注意力集中的	21	Friendly	友好的	38	Secure	安全放心的
5	Authentic	真材实料的	22	Genuine	真心真意的	39	Self-pleasing	取悦自己
6	Bored	无聊无趣的	23	Glad	乐意的	40	Simple	简单的
7	Carefree	无忧无虑的	24	Good	优质优等的	41	Steady	稳定可靠的
8	Cheap	便宜的	25	Happy	开心幸福的	42	Strong	劲头足的
9	Classy	有品位的	26	Impressive	印象深刻的	43	Sweet	甜蜜的
10	Comforting	舒服的	27	Interested	感兴趣的	44	Toneless	沉默的
11	Curative	治愈有疗效的	28	Intimate	贴心的	45	Traditional	传统的
12	Desire	想要的希望的	29	Joyful	令人欣喜的	46	Trustworthy	值得信赖的
13	Discontented	不满足的	30	Lonely	孤独孤单的	47	Unguilty	无负担/无负罪感的
14	Energetic	能量满满的	31	Mild	温和不强烈	48	Warm	温暖的
15	Enjoyable	享受的	32	Nostalgic	怀旧的	49	Whole	全面齐全的
16	Excited	令人兴奋的	33	Peaceful	平和宁静的	50	Youthful	青春年轻的
17	Fascinating	有吸引力的	34	Pleasant	令人愉快的			

**Table 4:** EFA factor dimensions, scale terms and factor loadings (n = 320).

Terms	Factor Loading				Common Loading
	Factor 1 (Property Cognition)	Factor 2 (Quality Perception)	Factor 3 (Acceptance Appraisal)	Factor 4 (Neutral Property Cognition)	
Energetic	0.778				0.614
Pleasant surprise	0.749				0.581
Impressive	0.731				0.593
Joyful	0.635				0.484
Sweet	0.585				0.509
Admiration	0.58				0.429
Interested	0.562				0.501
Active	0.545				0.481
Classy	0.505				0.501
Friendly	0.498				0.412
Desire	0.455				0.507
Curative	0.439				0.401
Amusement	0.415				0.355
Secure		0.785			0.666
Warm		0.695			0.534
Good		0.637			0.557
Trustworthy		0.634			0.546
Authentic		0.472			0.328
Genuine		0.414			0.358
Comforting			0.886		0.893
Relaxed			0.682		0.556
Self-pleasing			0.665		0.502
Unguilty			0.541		0.521
Glad			0.529		0.613
Happy			0.516		0.563
Enjoyable			0.469		0.539
Pleasant			0.463		0.523
Satisfied			0.409		0.417
Peaceful				0.756	0.63
Mild				0.694	0.496
Flat				0.652	0.576
Simple				0.607	0.466
Steady				0.487	0.375
Explanation of Variance	16.37%	13.64%	11.42%	9.89%	

**Table 5:** Inter-scale correlation of PDPES (n = 320).

Factors	Factor 1	Factor 2	Factor 3	Factor 4
Factor 1	1			
Factor 2	0.783 *	1		
Factor 3	0.832 *	0.886 *	1	
Factor 4	0.179	0.489 *	0.403 *	1

* Indicates a significant difference at *p* < 0.05.

**Table 6:** CFA results of P-PDPES and PDPES (n = 320).

	*n*	*χ*^2^/*df*	CFI	TLI	RMSEA	SRMR
P-PDPES	300	2.63	0.72	0.66	0.08	0.08
PDPES	320	2.16	0.95	0.93	0.07	0.04

**Table 7:** PDPES correlations and square root of AVE (n = 320).

Factors	Factor 1 (Property Cognition)	Factor 2 (Quality Perception)	Factor 3 (Acceptance Appraisal)	Factor 4 (Neutral Property Cognition)
Factor 1	0.655			
Factor 2	0.538	0.699		
Factor 3	0.624	0.664	0.566	
Factor 4	0.111	0.353	0.313	0.589

**Table 8:** PDPES reliability index.

	n	Factor 1 (Property Cognition)	Factor 2 (Quality Perception)	Factor 3 (Acceptance Appraisal)	Factor 4 (Neutral Property Cognition)	Total Score
Cronbach’s α	320	0.843	0.766	0.803	0.715	0.875
Reliability Composite	320	0.859	0.689	0.754	0.759	
Reset Reliability	50	0.676	0.602	0.703	0.618	0.663

**Table 9:** Emotion intensity of milk products using PDPES.

Factor	Sample	Mean Value	SE	95% CL	*p* Value
Factor 1 (Property Cognition)	total	3.26	0.04	3.18~3.33	**0.000**
	WM-4.0	3.34	0.07	3.21~3.48	
	WM-3.0	**3.02**	0.09	2.85~3.20	
	WM-LF	3.53	0.09	3.34~3.71	
	WM-P	3.23	0.08	3.08~3.39	
	LM	**3.16**	0.08	3.00~3.32	
Factor 2 (Quality Perception)	total	3.45	0.04	3.38~3.53	0.168
	WM-4.0	3.64	0.07	3.50~3.77	
	WM-3.0	3.42	0.09	3.23~3.60	
	WM-LF	3.39	0.11	3.18~3.60	
	WM-P	3.48	0.08	3.31~3.65	
	LM	3.34	0.08	3.17~3.51	
Factor 3 (Acceptance Appraisal)	total	3.41	0.04	3.33~3.49	**0.041**
	WM-4.0	3.56	0.08	3.40~3.71	
	WM-3.0	**3.12**	0.09	3.03~3.22	
	WM-LF	3.49	0.11	3.28~3.70	
	WM-P	3.43	0.09	3.24~3.61	
	LM	3.34	0.08	3.17~3.51	
Factor 4 (Neutral Property Cognition)	total	3.41	0.04	3.34~3.49	**0.000**
	WM-4.0	3.55	0.06	3.43~3.68	
	WM-3.0	3.49	0.08	3.32~3.66	
	WM-LF	**3.02**	0.10	2.83~3.22	
	WM-P	3.47	0.08	3.31~3.64	
	LM	3.52	0.07	3.38~3.66	

**Table 10:** Correlation between PDP Emotion Scale and the liking value of milk products.

Factor	WM-4.0	WM-3.0	WM-LF	WM-P	LM	Total
Factor 1 (Property Cognition)	0.695 *	0.776 *	0.779 *	0.799 *	0.792 *	0.786 *
Factor 2 (Quality Perception)	0.560 *	0.671 *	0.691 *	0.644 *	0.788 *	0.674 *
Factor 3 (Acceptance Appraisal)	0.751 *	0.849 *	0.845 *	0.860 *	0.820 *	0.839 *
Factor 4 (Neutral Property Cognition)	0.139	0.493 *	0.480 *	0.496 *	0.273 *	0.358 *
Total score	0.697 *	0.805 *	0.807 *	0.826 *	0.826 *	0.813

Note: * Indicates a significant difference at *p* < 0.05.

## Data Availability

The original contributions presented in this study are included in the article/Supplementary Materials. Further inquiries can be directed to the corresponding author.

## Supplementary Materials

The following supporting information can be downloaded at: https://www.mdpi.com/article/10.3390/foods14050827/s1, Figure S1: Example of questionnaire; Table S1: A list of emotion terms for pure dairy products; Table S2: The main components information of five dairy products (100 mL).
