# Supplementary material for: An Exploratory Study on the Development of a Pure Dairy Product Emotion Scale (PDPES): A Study of Milk Consumers in China
Source: Foods. 2025 Feb 27;14(5):827. doi: 10.3390/foods14050827 (PMC11898885; doi:10.3390/foods14050827)
Supplement: Supplementary file 1 [file foods-14-00827-s001.zip › foods-3469543-supplementary.pdf]

Supplementary Table S1 A list of emotion terms for pure milk products

No.	terms	No.	terms	No.	terms
1	Active	45	Exotic	89	Polite
2	Admiring	46	Fascinating	90	Powerful
3	Adventurous	47	Fashionable	91	Pretentious
4	Affectionate	48	Feminine	92	Professional
5	Afraid	49	Flat	93	Proud
6	Aggressive	50	Forthright	94	Quiet
7	Alert	51	Free	95	Refreshing
8	Amazed	52	Fresh	96	Relaxed
9	Amusement	53	Friendly	97	Relieve
10	Arrogant	54	Fun	98	Reputable
11	Ashamed	55	Genuine	99	Respective
12	Attentive	56	Glad	100	Romantic
13	Attractive	57	Good	101	Sad
14	Authentic	58	Good-natured	102	Satisfied
15	Balanced	59	Guilty	103	Scared
16	Beautiful	60	Happy	104	Secure
17	Bored	61	Healthy	105	Self-Pleasing
18	Calm	62	Homely	106	Self-Reward
19	Carefree	63	Hostile	107	Sensual
20	Cheap	64	Impressive	108	Serious
21	Childish	65	Indignant	109	Simple
22	Classical	66	Indulgent	110	Social
23	Classy	67	Innovative	111	Sophisticate
24	Comfortable	68	Insistant	112	Steady
25	Confident	69	Inspiring	113	Strong
26	Contempt	70	Interested	114	Sweet
27	Cool	71	Intimate	115	Tame
28	Curative	72	Irritating	116	Tedious
29	Daring	73	Jittery	117	Tender
30	Decompressed	74	Joyful	118	Tired
31	Depressed	75	Lonely	119	Traditional
32	Desire	76	Loving	120	Trustworthy
33	Determined	77	Masculine	121	Understanding
34	Disappointed	78	Mature	122	Unguilty
35	Discontented	79	Merry	123	Unhappy
36	Disgusted	80	Mild	124	Unique
37	Dissatisfied	81	Modern	125	Unpleasant Surprise
38	Distressed	82	Nervous	126	Upset
39	Eager	83	Nostalgic	127	Warm
40	Easygoing	84	Outdated	128	Whole
41	Energetic	85	Peaceful	129	Wild

42	Enjoyable	86	Pleasant	130	Worried
43	Enthusiastic	87	Pleasant Surprise	131	Youthful
44	Excited	88	Pleased		

Source: EsSense39®(39 terms), NMR(30 terms), EmoSensory®(19 terms), PrEmo®(14 terms),PANAS(20 terms) and collection from internet and industry survey (43 terms)

Supplementary Table S2 The main components information of five milk products (100 mL)

**Category**	**Code**	**Fat /g**	**Protein /g**	**Carbohydrate /g**	**Na^+ /mg**	**Ca^+ /mg**
Whole milk/UHT	WM-4.0	4.6	4.0	5.7	60	130
Whole milk/UHT	WM-3.0	3.7	3.0	4.8	60	100
Whole milk/UHT/UHT/lactose-free	WM-LF	3.6	3.0	4.9	67	100
Whole milk/Pasteurization	WM-P	4.2	3.6	5.3	60	120
Low-fat milk/UHT	LM	1.3	3.6	5.1	58	120

Please select the all the words which describe how you FEEL WHEN CONSUMING THE PURE MILK PRODUCTS

□ Secure	□ Polite	□ Self-pleasing	□ Mild	□ Irritating
□ Cheap	□ Warm	□ Enthusiastic	□ Modern	□ Jittery
□ Sweet	□ Mild	□ Steady	□ Modest	□ Joyful
□ Comforting	□ Whole	□ Strong	□ Balance	□ Lonely
□ Interested	□ Confident	□ Fascinating	□ Powerful	□ Enjoyable
□ Enthusiastic	□ Polite	□ Youthful	□ Irritating	□ Active

Supplementary Figure S1 Example of questionnaire
